# A long-term survivor of metachronous liver metastases of pancreatic serous cystic neoplasm associated with von Hippel–Lindau disease

**DOI:** 10.1186/s40792-021-01239-y

**Published:** 2021-06-30

**Authors:** Takashi Kokumai, Masamichi Mizuma, Katsuya Hirose, Hideaki Karasawa, Masaharu Ishida, Hideo Ohtsuka, Kei Nakagawa, Takanori Morikawa, Takashi Kamei, Atsushi Masamune, Toru Furukawa, Michiaki Unno

**Affiliations:** 1 Department of Surgery, Tohoku University Graduate School of Medicine, 1-1 Seiryomachi, Aobaku, Sendai, 980-8574 Japan; 2 Department of Investigative Pathology, Tohoku University Graduate School of Medicine, 1-1 Seiryomachi, Aobaku, Sendai, 980-8574 Japan; 3 Division of Gastroenterology, Tohoku University Graduate School of Medicine, 1-1 Seiryomachi, Aobaku, Sendai, 980-8574 Japan

**Keywords:** Serous cystadenocarcinoma, Serous cystic neoplasm, Von Hippel–Lindau disease, Metastasis

## Abstract

**Background:**

Pancreatic serous cystic neoplasm (SCN) is an uncommon exocrine neoplasm, which is believed to be a benign entity. However, some of these neoplasms may occasionally attain metastatic ability. Von Hippel–Lindau disease (VHL) manifests a dominantly inherited systemic syndrome accompanied by several benign or malignant tumors, including cystic tumors, in various organs. We describe here a long-term survival case who underwent surgical resection for metachronous liver metastases of pancreatic SCN associated with VHL disease.

**Case presentation:**

A 35-year-old woman with VHL underwent total pancreatectomy and right nephrectomy for pancreatic SCN and renal cell carcinoma, respectively. At the 4th year follow-up examination after the resection, contrast-enhanced computed tomography (CT) and gadolinium ethoxybenzyl diethylenetriamine penta-acetic acid (Gd-EOB-DTPA)-enhanced magnetic resonance imaging (MRI) showed arterially hyper-enhanced neoplastic lesions in the segment VI and VIII of the liver. Partial resections of the liver were performed 53 months after the initial surgery. At the 6th month follow-up examination from the second surgery, one and two tumors located in the liver segment III, and VIII, respectively, were detected by contrast-enhanced CT and Gd-EOB-DTPA-enhanced MRI. Anterior segmentectomy and partial resection of the segment III were performed 66 months after the initial surgery and 13 months after the second, respectively. The tumors were pathologically diagnosed as liver metastases of pancreatic SCN synonymous with serous cystadenocarcinoma. She remains disease-free without recurrence 6.5 years after the last operation.

**Conclusions:**

This is the first report of a case of metastatic SCN associated with VHL. Surgical resection might confer a favorable prognosis in patients of pancreatic SCN with liver metastases.

## Background

Pancreatic serous cystic neoplasm (SCN) is an uncommon tumor that accounts for 1−2% of exocrine neoplasms of the pancreas [1]. SCN, which is generally considered to be benign, is very rarely malignant. In 1989, George et al. reported the first autopsy case of malignant SCN of the pancreas metastasizing to the stomach and liver [2]. Since then several cases of malignant SCN have been reported. The actual proportion of malignancy in pancreatic SCNs has been reported to be only 0.6% [3].

Von Hippel–Lindau disease (VHL) is an autosomal dominant neoplasia syndrome that results from germline mutations in the *VHL* genes [4, 5]. These mutations lead to the development of several benign or malignant tumors, including cystic tumors, in various organs. Pancreatic SCNs associated with VHL were found in 9−11% of patients [6, 7]. However, no cases of malignant SCN associated with VHL have ever been reported.

We previously reported a case of VHL with a germline *VHL* mutation, 233 A > T, who underwent total pancreatectomy for multiple SCNs and right nephrectomy for renal cell carcinoma [8]. Herein, we report that this case developed multiple metachronous liver metastases of the SCN after the initial operation and underwent surgical resections for the metastases. This patient has been alive without any recurrence more than 6 years after the metastasectomies.

## Case presentation

**Fig. 1:**
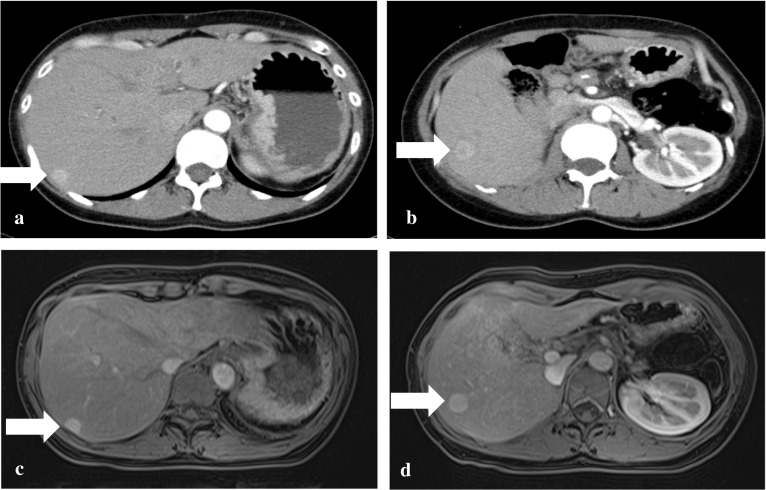
**a**, **b** Contrast-enhanced CT showed arterially hyper-enhanced lesions in liver segment VIII (**a**, arrow) and VI (**b**, arrow). **c**, **d** Gd-EOB-DTPA-enhanced MRI showed arterially hyper-enhanced lesions in liver segment VIII (**c**, arrow) and VI (**d**, arrow). *CT* computed tomography, *Gd-EOB-DTPA* gadolinium ethoxybenzyl diethylenetriamine penta-acetic acid, *MRI* magnetic resonance imaging

**Fig. 2:**
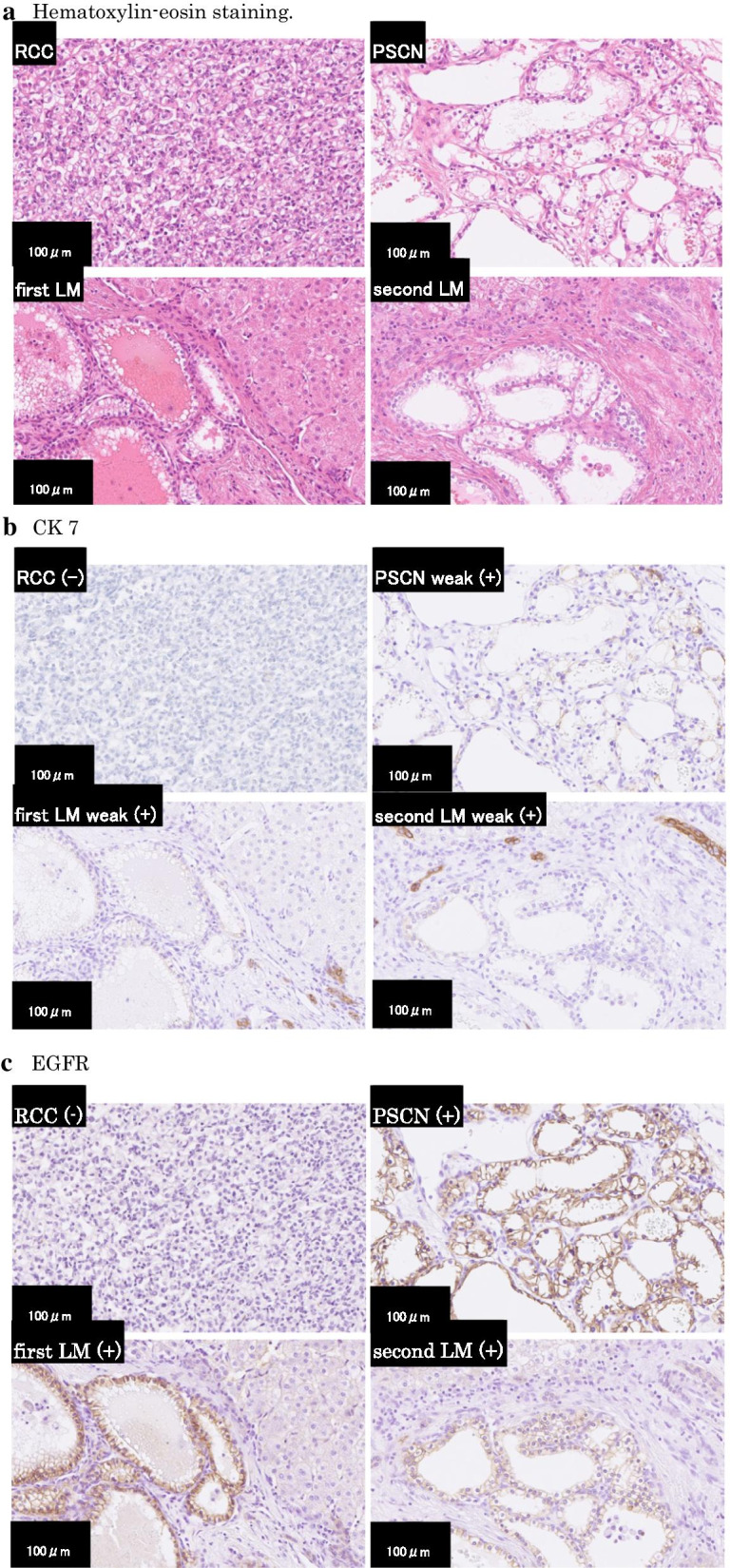

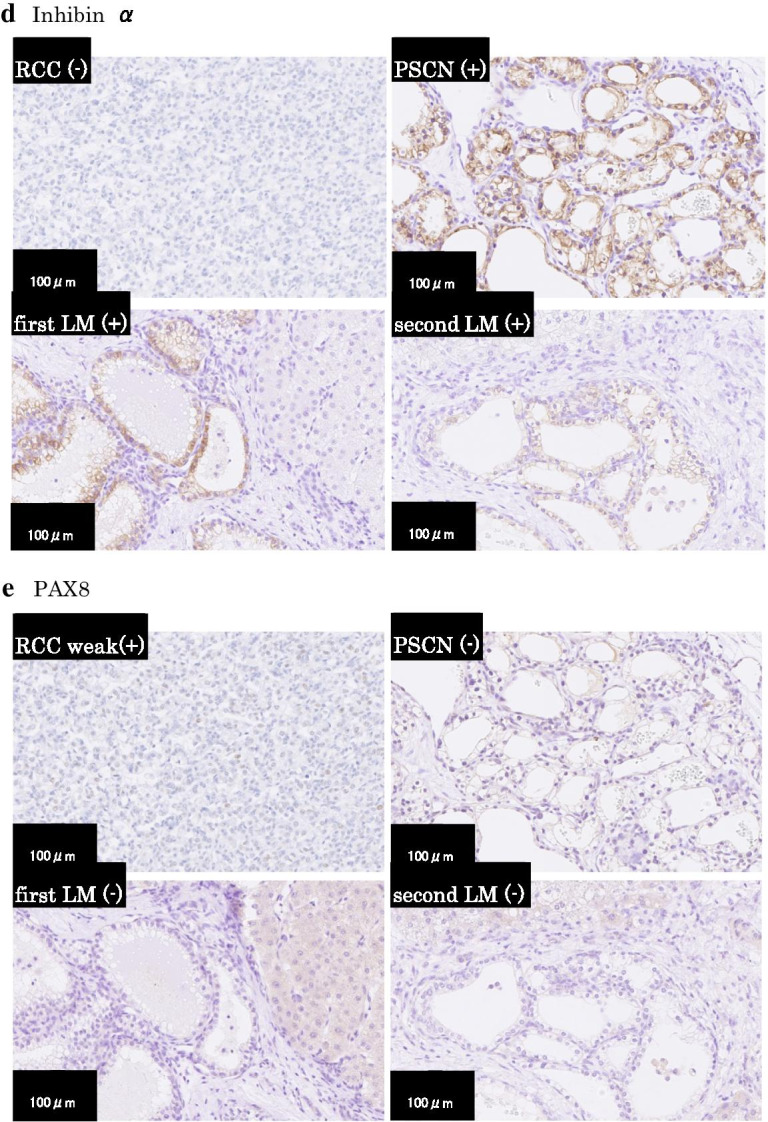
**a** Microscopic features of the primary renal cell carcinoma (RCC) and pancreatic serous cystic neoplasm (PSCN), and metachronous metastatic liver tumors (first LM and second LM). The metastatic liver tumors showed multiple microcysts consisting of cuboidal cells with clear cytoplasm in the portal regions. No involvement of the liver parenchyma was noted (hematoxylin and eosin stain, × 200). **b**–**e** Immunohistochemical analysis of CK7 (**b**), EGFR (**c**), inhibin α (**d**), and PAX8 (**e**) showed that PSCN and metastatic tumors (first LM and second LM) showed the same expression phenotypes that were different from those of RCC (× 200 in original magnifications)

A 35-year-old woman with VHL who underwent total pancreatectomy and right nephrectomy for pancreatic SCN and renal cell carcinoma (RCC), respectively, had postoperative follow-up examinations performed every 6 months [8]. At the 4th year follow-up examination, 12-mm and 15-mm tumors in the liver segment VIII and VI, respectively, were detected as hyper-enhanced mass in the early phase by contrast-enhanced computed tomography (CT) scan (Fig. 1a, b). Gadolinium ethoxybenzyl diethylenetriamine penta-acetic acid (Gd-EOB-DTPA)-enhanced magnetic resonance imaging (MRI) revealed arterially hyper-enhanced lesions in the liver, which were the same as the findings of the CT scan (Fig. 1c, d), showing iso-enhancement in the portal phase and hypo-enhancement in the delayed phase compared with the liver parenchyma. The liver tumors were radiographically diagnosed as metachronous metastases of pancreatic SCN or RCC. Partial resections of the liver were performed 53 months after the initial surgery. Contrast-enhanced intraoperative ultrasonography (CE-IOUS) using Sonazoid (gaseous perflubutane) showed early enhanced hyperechoic tumors in the liver, whereas intraoperative indocyanine green (ICG) fluorography showed no liver tumor. In the intraoperative macroscopic findings, the liver tumors were obscure. In microscopical observations, the tumors were confined in portal regions without invasion into the hepatic parenchyma, which consisted of microcysts lined by cuboidal epithelial cells with clear cytoplasm containing large amounts of glycogen that were stained by periodic acid Schiff with abolishment by diastase. Furthermore, immunohistochemically, the tumor was positive for pan-cytokeratin (AE1/AE3), epithelial membrane antigen (EMA), cytokeratin (CK) 7, CK18, CK19, epidermal growth factor receptor (EGFR), inhibin α, laminin, type IV collagen, neuron specific enolase (NSE), and mucin (MUC) 6, but negative for CK20, CA125, S-100, CD10, CA19-9, paired-box gene (PAX) 8, vimentin, CD56, MUC1, MUC2, and MUC5AC. These immunohistochemical findings were similar to those of the primary pancreatic SCN, but differed from the RCC resected previously (Fig. 2). Thus, the tumors were pathologically diagnosed as liver metastases of the pancreatic SCN, synonymous with serous cystadenocarcinoma in the World Health Organization (WHO) Classification of Tumours of the pancreas [9].

**Fig. 3:**
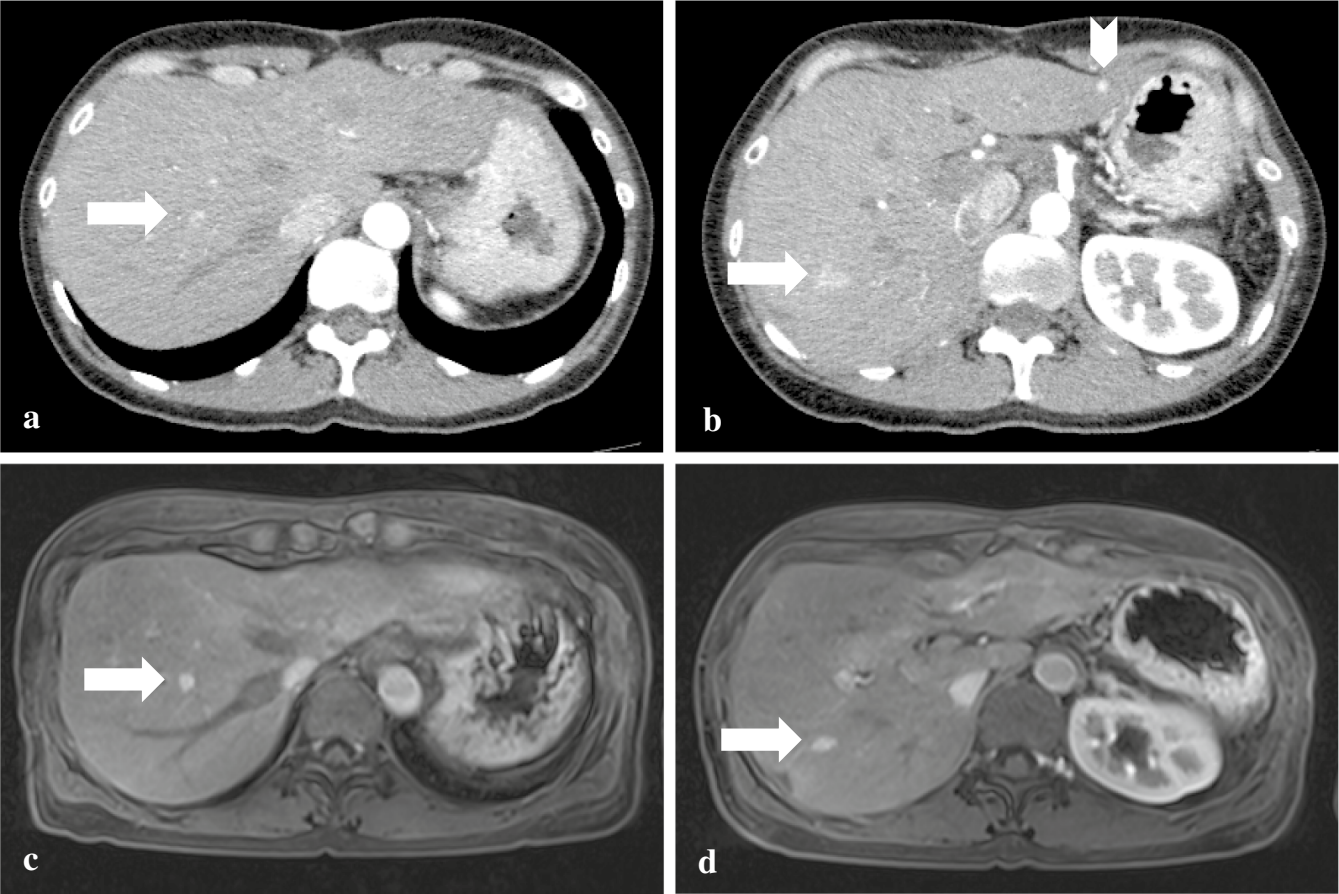
**a**, **b** Contrast-enhanced CT scan. Post-arterially hyper-enhanced lesions were revealed in the liver segment VIII (**a**, arrow), segment III (**b**, arrowhead), and VIII (**b**, arrow). **c**, **d** Gd-EOB-DTPA-enhanced MRI. Two arterially hyper-enhanced lesions were revealed in liver segment VIII (**c** and **d**, arrow). The lesion of segment III detected by the CT scan was undetectable. *CT* computed tomography, *Gd-EOB-DTPA* gadolinium ethoxybenzyl diethylenetriamine penta-acetic acid, *MRI* magnetic resonance imaging

After the second surgery, the patient had follow-up examinations performed every 6 months again. At the first follow-up examination, one and two tumors located in the liver segment III and VIII, respectively, were detected by contrast-enhanced CT and Gd-EOB-DTPA-enhanced MRI (Fig. 3). Compared to the contrast-enhanced CT scan, the Gd-EOB-DTPA-enhanced MRI more clearly showed the tumors of segment VIII (Fig. 3a, c), as in the previous examination. On the other hand, the tumor of segment III was clearly detected by the contrast-enhanced CT, though undetectable in the Gd-EOB-DTPA-enhanced MRI (Fig. 3b, d). Liver metastases of SCN were radiologically suspected. Since it was a short period since the hepatectomy, radiological follow-up examinations were additionally performed every three months to observe changes in the number and size of the tumors. No increase in the number of the tumors was seen in the radiological examinations after 6 months. Thus, the liver tumors were considered to have a surgical indication. Anterior segmentectomy and partial resection of segment III were performed 66 months after the initial surgery and 13 months after the second, respectively. Findings of CE-IOUS using Sonazoid and intraoperative ICG fluorography were similar to those in the second surgery. Pathologically, the resected tumors again showed morphological and immunohistochemical features similar to the primary pancreatic SCN, hence these tumors were pathologically diagnosed as multiple metachronous liver metastases of pancreatic SCN, synonymous with serous cystadenocarcinoma (Fig. 2). Although the radiological findings of CT/MRI in the tumor of segment III were different from those in the tumors of segment VIII, no histopathological findings suggesting a difference in the radiological findings were observed.

Since then, radiological follow-up examinations have been continuously performed. The patient has remained disease-free without any recurrence after the third operation for 6.5 years until the recent last follow-up.

## Discussion

To the best of our knowledge, we presented the first case of liver metastases of VHL-associated SCN. In addition, this case had a long-term fair prognosis without any recurrence after two hepatectomies for multiple metachronous liver metastases. Points mainly discussed in the clinical course of this case includes: (1) the clinical features of the distant metastasis of SCN; (2) treatment methods and prognosis regarding metastatic SCN, and (3) follow-up for SCN including VHL-associated SCN.

Metastasis of SCN is extremely rare. According to the current WHO classification, serous neoplasms of the pancreas are classified into serous cystadenoma and serous cystadenocarcinoma, in which “the diagnosis of malignancy in pancreatic serous neoplasms is restricted to cases with unequivocal distant metastasis beyond the pancreatic/peripancreatic bed.” It was also described that < 20 cases of serous cystadenocarcinoma have been reported when strict criteria requiring the presence of true distant metastases are used. Consistent with this description, when we conducted a literature review of pancreatic serous cystadenocarcinoma using PubMed search using the keywords “pancreas” and “serous cystadenocarcinoma”, we found 16 cases of pancreatic serous cystadenocarcinoma, as reviewed in Table 1. There have been no reports of metastatic SCN associated with VHL. Regarding metastasis, liver metastases were found in all patients. Multiple liver metastases were observed in 81% (13/16) of the reviewed cases. There were two cases with metastases in multiple organs: one case revealed metastases to the liver, lung, bone and adrenal glands, and the other case revealed liver metastasis and peritoneal dissemination. Synchronous metastases were seen in 50% of the reviewed cases, and the remaining 50% were metachronous metastasis. With respect to the treatment and the prognosis for metastatic SCNs, all of reported three cases with single liver metastasis underwent surgical resection, and one of them was alive without recurrence 3 years after surgery, although the long-term prognosis of the remaining two cases was unknown. Multiple liver metastases were reported in 13 cases. Three cases underwent radical resection, and were alive without recurrence 1–3 years after surgery. Locoregional therapy, microwave coagulo-necrotic therapy (MCN) or radiofrequency ablation (RFA), was performed in three cases, in one of which the patient was alive with no recurrence one year after MCN. The remaining two cases had liver recurrences. The case that underwent MCN for initial liver metastases received partial resection and repeated MCN for new liver metastases after 9 years, and has been alive without any recurrences one year after the second surgery. On the other hand, the case that underwent RFA for initial liver metastases received MCN for new liver metastases after 4.5 years, had multiple liver metastases again 15 months after second surgery, and has been alive 6 years after the initial metastasis with liver metastases observed because of old age. Although these two cases were treated with systemic chemotherapy, one showed no change in the metastatic tumors, and another treated with sunitinib had unknown efficacy for the metastatic tumors. Including our case, metastasectomy or locoregional therapy is suggested to have favorable efficacy for liver metastases of pancreatic SCN.

**Table 1:** Characteristics of serous cystadenocarcinoma of the pancreas

Case	Author	Year	Age/sex	Size (cm)	Signs or symptoms	Metastases (single/multiple liver metastases)	Synchronous/metachronous (time interval from initial treatment)	Treatment for metastases	Outcome (time after diagnosis of metastases)
1	George et al. [2]	1989	70/M	11	Hemorrhage from gastric varices	Liver (multiple)	Synchronous	None	Intraoperative death due to blood loss
2	Friedman et al. [13]	1990	74/F	19	Right flank pain, weight loss, abdominal mass	Liver (multiple), lung, bone, adrenal glands	Synchronous	None	Dead of primary disease
3	Okada et al. [14]	1991	63/F	12	Abdominal pain	Liver (multiple)	Metachronous (4 years)	Partial hepatectomy	Alive (NR) (1 year)
4	Yoshimi et al. [15]	1992	63/F	12	abdominal mass, epigastric pain	Liver (multiple)	Metachronous (3 years)	Partial hepatectomy	Alive (NR) (3 years)
5	Ishikawa et al. [16]	1998	63/F	12	Abdominal pain	Liver (single)	Metachronous (3 years)	Partial hepatectomy	Not described
6	Eriguchi et al. [17]	1998	56/F	16	Abdominal mass	Liver (multiple)	Synchronous	MCN	Liver recurrence 9 years after MCN Alive (NR) 1 year after hepatectomy and repeated MCN for liver recurrence (10 years after initial metastasis)
7	Wu et al. [18]	1999	57/F	No data	Incidental finding	Liver, (multiple) peritoneum	Metachronous (10 years)	None	Not described
8	Strobel et al. [19]	2003	56/F	14	Abdominal pain, diarrhea, weight loss	Liver (single)	Metachronous (3 years)	Partial hepatectomy	Alive (NR) (3 years)
9	Franko et al. [20]	2008	68/F	5	Hemorrhage from gastric varices, abdominal pain, weight loss	Liver (multiple)	Metachronous (3 years)	None	Dead of primary disease (9 months)
10	Bano et al. [21]	2011	62/M	7	Abdominal pain, vomiting, weight loss	Liver (multiple)	Metachronous (1 year)	MCN	Alive (NR) (1 year)
11	Bramis et al. [22]	2012	86/F	17	Abdominal pain	Liver (multiple)	Synchronous	None	Dead of unrelated disease (1 month)
12	Wasel et al. [23]	2013	68/M	12	Incidental finding	Liver (multiple)	Synchronous	4 months of chemotherapy	Alive (1 year)
13	Kainuma et al. [24]	2015	69/M	6	Abdominal discomfort	Liver (multiple)	Synchronous	Partial hepatectomy	Alive (NR) (2.5 years)
14	Huh et al. [25]	2016	52/F	9	Abdominal mass	Liver (multiple)	Metachronous (5 years)	None	Alive (1.5 years)
15	Van Dyke et al. [26]	2016	78/M	16	Incidental finding	Liver (multiple)	Synchronous	RFA	MCN for liver recurrence 4.5 years after RFA Alive observing liver recurrence 15 months after MCN (6 years after initial metastasis)
16	Massaras et al. [27]	2020	60/F	9	Incidental finding	Liver (single)	Synchronous	Sunitinib (4 cycles), partial hepatectomy	Alive (NR) (3 years)

*F* female, *M* male, *MCN* microwave coagulo-necrotic therapy, *NR* no recurrence, *RFA* radiofrequency ablation

The time interval between detection of pancreatic SCN and that of metachronous metastases was from 1 to 10 years. Similar to our case, 75% (6/8) of metachronous metastatic cases were burdened with distant metastases 3–5 years after the detection of pancreatic SCN. Interestingly, late metastatic relapses due to a relatively indolent course were detected 9 and 10 years after the initial treatment. Therefore, although there are concerns about the cost–benefit performance and radiological exposure due to radiological examination, the long-term and continuing follow-up might be needed after the treatment of pancreatic SCN. In addition, in cases of VHL-associated SCN, we should be careful about the onset of other neoplastic diseases, such as hemangioblastoma of central nervous system, RCC and pheochromocytoma [10–12].

Interestingly, the histopathological findings of our case showed that the metastatic tumors in the liver were localized in portal regions, and revealed no proliferation or infiltration into the liver parenchyma. No similar findings have been reported in cases with liver metastases of SCN. Further accumulation of similar cases is needed to investigate whether this is characteristic histopathological findings of liver metastases of pancreatic SCN associated with VHL disease.

## Conclusion

In summary, a long-term survivor of metachronous liver metastases of pancreatic SCN associated with VHL disease was presented. This is the first report of metastatic SCN associated with VHL. A long-term follow-up may be needed for SCN. Surgical resection might confer a favorable prognosis in cases with liver metastases of pancreatic SCN.

## Data Availability

The datasets supporting the conclusions of this article are included within the article.

## Abbreviations

SCN: Serous cystic neoplasm
VHL: Von Hippel–Lindau disease
CT: Computed tomography
RCC: Renal cell carcinoma
Gd-EOB-DTPA: Gadolinium ethoxybenzyl diethylenetriamine penta-acetic acid
MRI: Magnetic resonance imaging
CE-IOUS: Contrast-enhanced intraoperative ultrasonography
ICG: Indocyanine green
EMA: Epithelial membrane antigen
CK: Cytokeratin
EGFR: Epidermal growth factor receptor
NSE: Neuron-specific enolase
MUC: Mucin
CD: Cluster of differentiation
PAX: Paired-box gene
WHO: World Health Organization
MCN: Microwave coagulo-necrotic therapy
RFA: Radiofrequency ablation
